# Supplementary material for: Amorphous Pterostilbene Delivery Systems Preparation—Innovative Approach to Preparation Optimization
Source: Pharmaceutics. 2023 Apr 13;15(4):1231. doi: 10.3390/pharmaceutics15041231 (PMC10145601; doi:10.3390/pharmaceutics15041231)
Supplement: Supplementary file 1 [file pharmaceutics-15-01231-s001.zip › pharmaceutics-2282946-supplementary.pdf]

pharmaceutics

MDPI

Supplementary Materials

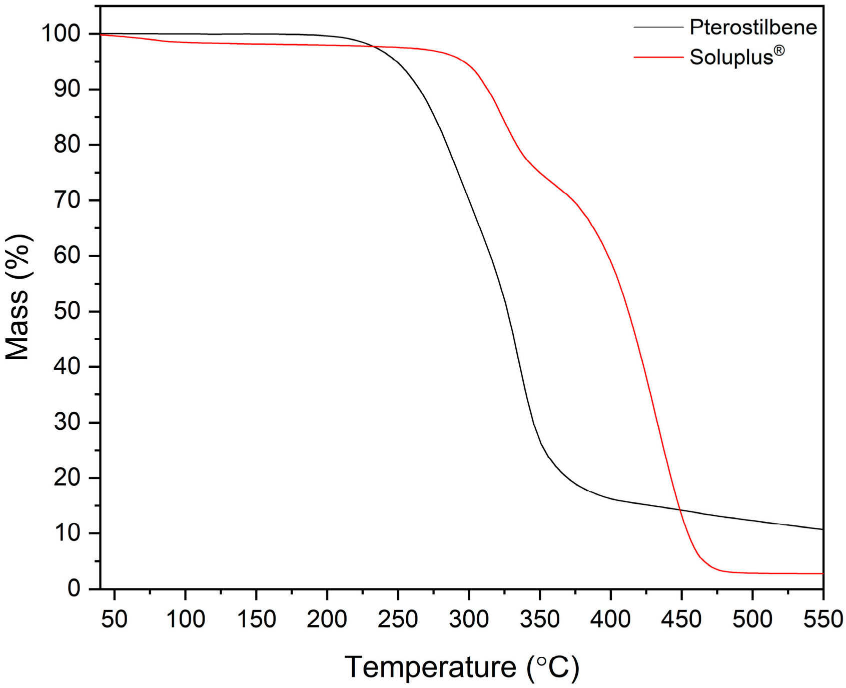

Figure S1. TG analysis of pterostilbene and Soluplus®.

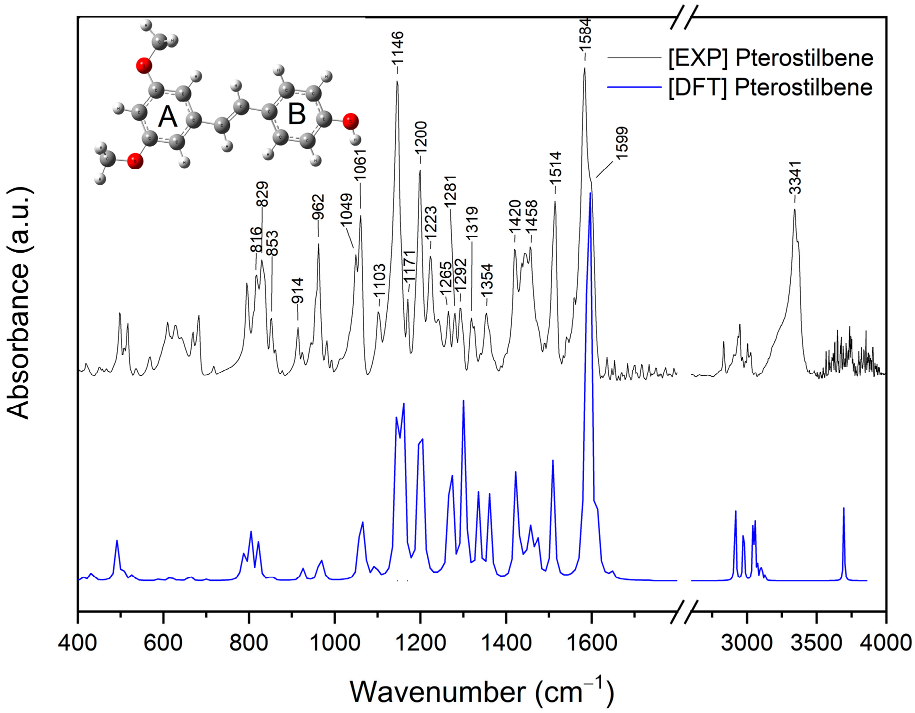

Figure S2. Experimental (EXP) and calculation (DFT) IR absorption spectra of pterostilbene at room temperature.

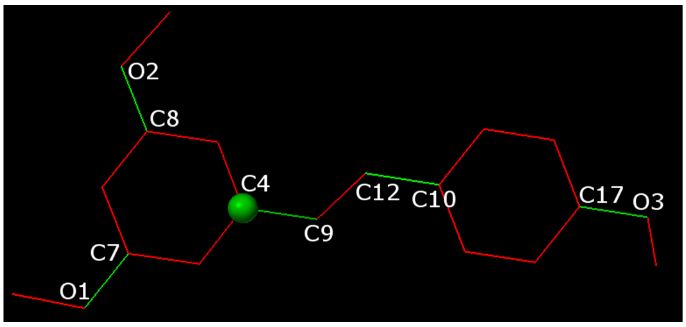

Figure S3. Torsion tree of pterostilbene. The “root” of the torsion tree is shown as a green sphere. The rotatable and non-rotatable bonds are shown as a green line and red line, respectively.

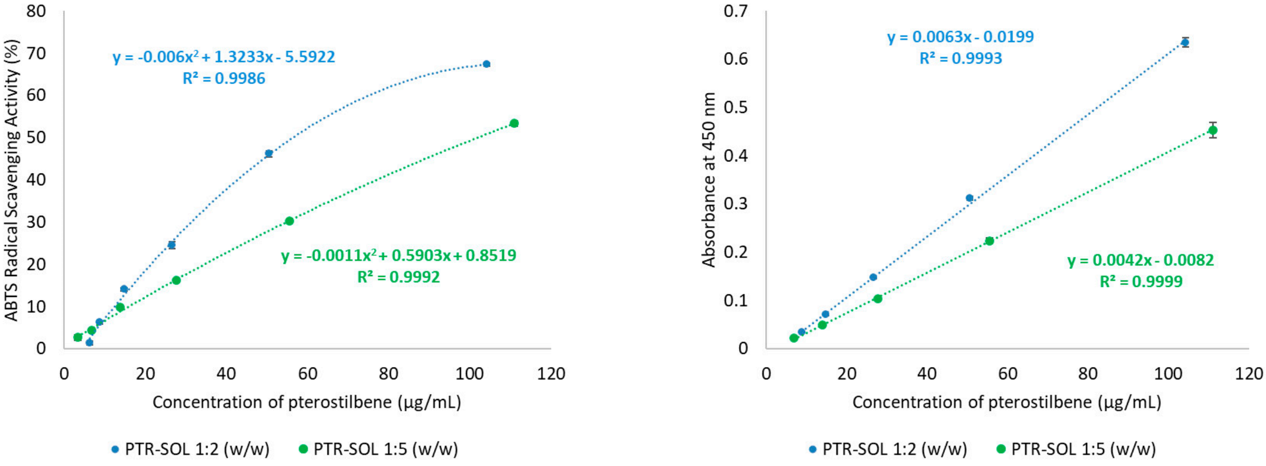

(a) (b)

Figure S4. Antioxidant activity of PTR-SOL 1:2 *w/w* and PTR-SOL 1:5 *w/w* (a) ABTS radical scavenging activity in relation to the pterostilbene concentration; (b) Cupric ion (Cu^{2+}) reducing activity in relation to the pterostilbene concentration.

Table S1. Selected characteristic theoretical (DFT) and experimental (EXP) bonds (in cm^{-1}) of pterostilbene (PTR). Assignments of PTR bands made based on DFT calculations with application of 6-31G(d,p) basis set.

Pterostilbene		Band assignment
**DFT**	**EXP**	
787	816	2–CHw + CHt at benzene ring B
805	829	CHt at benzene ring A + 4'– ang 6'–CHw
822	853	6–, 5'– and 6'–CHw
926	914	5' and 6'–CHt
970	962	CHw at main chain
1066	1049<br>1061	C–C–Cs in benzene ring A +2–, 4–, 6–CHb + 5–OCs
1092	1103	2–, 4–CHb + 3–OCs
1144	1146	2– and 6–CHb
1161	1171	2'– and 5'–CHb + 4'–OHb
1205	1200<br>1223	3– and 5–COCas
1205	1265	2–, 4–, 6–CHb + CHb at main chain + 2'–, 3'–CHb
1301	1281<br>1292	C–C–Cb in benzene ring A
1335	1319	2'– 5'–, 6'–CHb + C–C–Cas in benzene ring B + 4'–OHb
1362	1354	CHw in 3– and 5–OCH_3 group + def. benzene ring A + CHb at main chain
1422	1420	CHw in 3– and 5–OCH_3 group + 2–, 4–, 6–CHb
1475	1458	CHb and CHw in 3– and 5–OCH_3 group
1509	1514	2'–, 3'–, 5'– and 6'–CHb + C–C–Cs in benzene ring B
1597	1584	2–, 4–, 6–CHb + C–C–Cas in benzene ring A
1614	1599	C=Cs in in benzene ring B
2919	2853	CH_3s in 5–OCH_3 group
2972	2942	CH_2s in 5–OCH_3 group
3041	3005	CH_3s in 3–OCH_3 group
3059	3028	CHs at benzene ring B and at main chain
3694	3341	4'–OHs

Legend: s - stretching, t - twisting, w - wagging, def. – deformation. Carbon atom numbers and localization of benzene ring A and B were described in Figure 6a.

Table S2. Selected characteristic bonds (in cm^{-1}) of pterostilbene (PTR), Soluplus® (SOL), and systems of PTR-SOL (ratio 1:2, and 1:5). Assignments of PTR bands made based on DFT calculations, and SOL based on literature.

PTR	SOL	PTR-SOL 1:2 w/w	PTR-SOL 1:5 w/w	Band assignment
816		–	–	2–CHw + CHt at benzene ring B
829		–	–	CHt at benzene ring A + 4'– ang 6'–CHw
	841	835	839	*
853		–	–	6–, 5'– and 6'–CHw
914		–	–	5' and 6'–CHt
962		↓	–	CHw at main chain
	974	–	↓	*
	1020	↓	↓	*
1049		↓	–	C–C–Cs in benzene ring A +2–, 4–, 6–CHb + 5–OCs
1061		1063↓	–	
1103		↓	–	2–, 4–CHb + 3–OCs
1146		1148↓	1150↓	2– and 6–CHb
1171		↓	↓	2'– and 5'–CHb + 4'–OHb
	1196	1200	1200	
1200		–	–	3– and 5–COCas
1223		–	–	
	1234	↓	↓	C–O–C s in the ether groups [1]
1265		↓	–	2–, 4–, 6–CHb + CHb at main chain + 2'–, 3'–CHb
1281		↓	–	C–C–Cb in benzene ring A
1292		↓	–	
1319		–	–	2'– 5'–, 6'–CHb + C–C–Cas in benzene ring B + 4'–OHb
1354		–	–	CHw in 3– and 5–OCH_3 group + def. benzene ring A + CHb at main chain
1420		–	–	CHw in 3– and 5–OCH_3 group + 2–, 4–, 6–CHb
	1420	1423	1422	*
	1437	1443	1441	*
1458		–	–	CHb and CHw in 3– and 5–OCH_3 group
	1458	↓	↓	*
	1475	1477↓	1477↓	C–O–C s in the ether groups [1]
1514		↓	↓	2'–, 3'–, 5'– and 6'–CHb + C–C–Cs in benzene ring B
1584		1586↓	1587↓	2–, 4–, 6–CHb + C–C–Cas in benzene ring A
1599		+	+	C=Cs in in benzene ring B
	1634	↓	↓	C=O stretching in tertiary amide [2,3] in the caprolactam [4] or C(O)N [5]
	1734	↓	+	C=O stretching in the ester [2,3] or OC(O)CH3 [5]
2833		2837↓	–	CH_3s in 5–OCH_3 group
	2859	↓	+	C–H stretching [3]
	2926	2930	↑	aliphatic –CH stretching [3]
2949		–	–	CH_2s in 5–OCH_3 group
3005		–	–	CH_3s in 3–OCH_3 group
3028		–	–	CHs at benzene ring B and at main chain
3341		+	–	4'–OHs
3370		+	–	

Legend: ↓ - decrease in peak intensity, ↑ - increase in peak intensity, + - a band is observed in this range, – - a band is not observed in this range, * - no information in the literature, s - stretching, t - twisting, w - wagging, def. – deformation. Carbon atom numbers and localization of benzene ring A and B were described in Figure 6a.

Table S3. Validation parameters of HPLC-DAD methods for concentration determination of pterostilbene

Pterostilbene	
Parameter	PTR dissolved in acetonitrile; Injection volume 10 µL
Linearity range (mg·mL^{-1})	0.002-0.0006
Correlation coefficient (r)	1.00
a ± S_a	16 778 474 262.6 ± 183692326.8
b ± S_b	- 687 544.89 ± 227956.1827 insignificant (α=0.05)
LOD (mg·mL^{-1})	0.000060
LOQ (mg·mL^{-1})	0.00020
Retention Time	4.275
